# Effectiveness of Gamification on Enjoyment and Satisfaction in Older Adults: Systematic Review and Meta-Analysis

**DOI:** 10.2196/72559

**Published:** 2025-06-12

**Authors:** Javier Bravo-Aparicio, Iria Trillo-Charlín, Juan Avendaño-Coy, Hector Beltran-Alacreu

**Affiliations:** 1 Toledo Physiotherapy Research Group, Faculty of Physiotherapy and Nursing of Toledo, Universidad de Castilla-La Mancha, Av Carlos III sn, Toledo, 45071, Spain, 34 925268800; 2 Toledo Physiotherapy Research Group, Instituto de Investigación Sanitaria de Castilla-La Mancha, Castilla-La Mancha, Spain

**Keywords:** gamification, exergaming, enjoyment, satisfaction, older adults

## Abstract

**Background:**

Sedentary behavior is highly prevalent among older adults, with adherence to exercise being a major challenge. Exercise offers substantial physical, psychological, and social benefits, but enjoyment is a key factor influencing adherence. Technology-based interventions have shown promise in enhancing motivation and participation, demonstrating higher adherence rates than conventional treatments, although challenges such as motivation loss and technological barriers persist. This review evaluates the effectiveness of active video game interventions on enjoyment and satisfaction in older adults.

**Objective:**

This systematic review and meta-analysis aims to determine whether active video games are superior to other interventions in generating greater enjoyment or satisfaction in older adults.

**Methods:**

PubMed, Cochrane, PEDro, SPORTDiscus, CINAHL, Web of Science, and Scopus databases were searched from inception to September 30, 2024, to identify randomized clinical trials or crossover studies. The primary outcome was enjoyment or satisfaction, assessed using various scales, including the Physical Activity Enjoyment Scale, Intrinsic Motivation Inventory, User Satisfaction Questionnaire, and Likert-type scoring scales. Secondary outcomes included adherence rates and adverse effects. Cochrane Risk of Bias 2 tool was used to evaluate the risk of bias.

**Results:**

Five studies were included in the quantitative analysis. The results indicated a significant improvement in enjoyment or satisfaction compared to the control groups (standardized mean difference 0.34, 95% CI 0.05-0.64; *P*=.02; *I*^2^=24%), although the effect size was small. Secondary outcomes could not be analyzed due to insufficient data in the selected studies.

**Conclusions:**

Active video game interventions may improve enjoyment and satisfaction in older adults, but the evidence remains of low certainty.

## Introduction

The global population of older adults is projected to surpass 994 million by 2030, and this trend is expected to continue in the following years [1]. A major concern in aging is the reduced expectancy of healthy life years. The 3 aspects of healthy or successful aging are cognitive or mental well-being, social fulfillment, and physical health, with exercise being crucial for physical health [2]. However, older adults are among the most sedentary population groups, as sedentary behavior seems to increase with age, with older adults spending 62% to 86% of the day in sedentary behavior [3-5].

In 2018, the World Health Organization (WHO) launched an intervention plan aimed at reducing physical inactivity among adults and adolescents by 15% by 2030 [6]. This initiative enhances individual and community health by creating active societies, environments, people, and systems. The intervention emphasizes the importance of exercise or physical activity as a primary treatment approach, given its numerous benefits and low risks of side effects. Regular physical activity improves mortality rates, life expectancy, and physical and functional health outcomes [7-12].

There are discrepancies in the literature regarding the risks and benefits of sedentary behavior and physical activity on physical, psychological, and social outcomes [13,14]. Consequently, sedentary lifestyles remain prevalent among older populations, as barriers or facilitators to physical activity adherence arise from intrapersonal factors (physical and mental health and individual preferences), interpersonal influences, as well as physical, structural, and organizational environments [15]. Numerous barriers and facilitators affect older adults’ engagement and adherence to exercise, shaped by individual experiences and preferences. Researchers have suggested that fun should be incorporated into the FITT (Frequency, Intensity, Time, Type) prescription model [16], as enjoyment may be a critical factor in exercise adherence. Studies have shown that patients perceive exercise differently—some view it as a pleasant activity, while others regard it as an obligation like taking medicine [15,17]. Addressing this barrier through an immediate reward system like enhancing enjoyment could positively transform patients’ exercise experiences and potentially improve adherence to physical activity interventions [18,19]. This can be explained through various theoretical frameworks such as operant conditioning theory, self-determination theory, or Ekkekakis model, which link enjoyment to perceived exertion [20,21]. Technology-based interventions, particularly those that integrate engaging and interactive elements, offer a promising solution to enhance motivation, make exercise more enjoyable, and encourage sustained participation among older adults [19].

Gamification, which applies video game design elements such as points, badges, leaderboards, and avatars in nongame contexts, has become an increasingly popular tool in recent years for enhancing adherence to various interventions. Gamification may positively influence user behavior and experience, although its effectiveness may vary depending on the intervention, as inconsistent results have been reported across different age groups [22,23]. Video games or technology-based interventions have demonstrated adherence rates as high as 91%, and in some cases, rates up to 1.38 times higher than conventional exercise treatments or no intervention, which could suggest that greater adherence to physical activity might lead to enhanced health benefits [19,24,25]. Adherence rates in exercise programs for older adults range from 65% to 86% but tend to decline in unsupervised training programs or when the duration exceeded 12 weeks, suggesting that factors such as supervision, program length, and the engaging nature of the intervention play a crucial role in maintaining adherence [19,24,26]. Despite these benefits, such interventions also present challenges, including loss of motivation or interest, space limitations, technological barriers, and feelings of embarrassment when using video games [24,27].

This systematic review aims to primarily evaluate the satisfaction and enjoyment experienced by older adults through active video games. The secondary objective was to determine their adherence to treatment and the possible side effects of the intervention. These metrics are essential for understanding intervention efficacy and long-term adherence.

## Methods

### Study Registration

The protocol for this systematic review and meta-analysis was registered in PROSPERO (CRD42024593212). This analysis was conducted following the recommendations of the Cochrane Collaboration and the PRISMA (Preferred Reporting Items for Systematic Reviews and Meta-Analyses) guidelines.

### Search Strategy

A bibliographic search was completed between September 21 and 30, 2024, in the following health and sports science databases: PubMed, Cochrane, Web of Science, CINAHL, SPORTDiscus, PEDro, and Scopus. The search strategies used are available in Multimedia Appendix 1.

### Inclusion and Exclusion Criteria

The inclusion criteria for this review were as follows: (1) randomized clinical or controlled trials and crossover studies; (2) patients older than 60 years; (3) exercise or physical activity intervention using gamification, including commercial apps, exergames, or serious games; (4) the comparison group had to perform some form of active exercise, follow their usual treatment, or do nothing; and (5) use of enjoyment or satisfaction scales.

Studies that met any of the following criteria were excluded: (1) case series, observational studies, and conference proceedings and (2) use of enjoyment or satisfaction scales in only one of the groups. No language exclusion criteria were applied.

### Study Selection

Studies were selected based on a predefined PICOS (Population, Intervention, Comparison, Outcome) framework established at the outset of the review. The population included older adults, the intervention involved exercise delivered through video games, and the outcome focused on assessing the levels of enjoyment or satisfaction. After defining the search strategy, studies were entered into Rayyan (an app) [28] to exclude duplicate papers. Two researchers selected the studies according to the inclusion and exclusion criteria; in case of disagreement, a third researcher reviewed the study until a consensus was reached.

### Data Extraction

First, 2 reviewers extracted informative data from the studies independently; in case of discrepancies, a third reviewer resolved this. The data to be extracted were first author and year, number of participants, design, groups, type of intervention in both groups, outcomes, number of sessions, session time, perceived effort, hardware and software used, and follow-up.

The second part consisted of extracting data values for the different outcomes—both primary and secondary. For the primary outcome of exercise enjoyment or satisfaction and for the secondary outcomes of adherence and adverse effects, mean and standard deviation values were extracted. When values were reported as change or as final values, the extraction of the final values for the analysis was determined as the preferred option. If data were only available in graphs, the graph digitization software GraphGrabber version 2.0.2 [29] was used for extraction.

### Risk of Bias

The methodological quality of the included studies was assessed by 2 independent reviewers (JBA and ITC) using the Cochrane Risk of Bias 2 tool, which evaluates the possible risk of bias in randomized trials for both parallel and crossover design studies [30]; in case of disagreement, the third reviewer broke the tie (HBA). This scale assesses bias based on 5 domains: process randomization, missing data on outcomes, outcome measurement, selection of reported outcomes, and deviations from intended interventions. An additional domain, bias arising from period and carryover effects, was assessed in crossover studies.

The GRADE (Grades of Recommendation Assessment Development and Evaluation) rating system was used to assess the quality of evidence. Publication bias was also assessed using the funnel plot and Egger test for publication asymmetry.

### Main Outcomes

The primary outcome variable for this review was exercise enjoyment or satisfaction, which was assessed using the scales reported in the included studies. These scales were not predetermined but were identified during the review process based on the methodologies of the selected studies. Each scale was included because it was used by the respective studies to measure enjoyment or satisfaction, ensuring consistency with their reported outcomes. The following tools were identified.

1. Physical Activity Enjoyment Scale: This scale is a validated and reliable tool used to assess the level of enjoyment individuals experience during a physical activity. The studies reviewed utilized a modified 5-item version of the scale, with responses recorded on a Likert scale ranging from 1 to 7 [31].
2. Intrinsic Motivation Inventory: This is a multidimensional scale designed to assess intrinsic motivation, with various subscales, including interest or enjoyment [32]. Only the interest or enjoyment subscale was used as an outcome measure in the study reviewed.
3. User Satisfaction Questionnaire: This is a 15-item questionnaire divided into 2 parts, that is, the benefits and pitfalls of the intervention and self-perceived improvements in physical and cognitive outcomes.

Two studies [33,34] did not use specific enjoyment or satisfaction scales. Instead, the participants were directly asked about their levels of enjoyment or satisfaction, and their responses were measured using Likert-type scales.

### Statistical Analysis

We assessed the overall effects of exercise through video games on enjoyment or satisfaction in older adults. As secondary outcomes, the effects of gamification compared to those of other interventions on adherence and adverse effects were analyzed. Subgroup analyses of the primary outcome (exercise enjoyment or satisfaction) were conducted to explore the key variables potentially influencing variations in enjoyment. These included session time (<10 min vs >10 min), target population (older adults without reported health conditions vs older adults with reported health conditions), immersion type (virtual reality vs augmented reality), type of control group (active vs passive), and the number of sessions (1 session vs >10 sessions).

The inverse variance method analyzed the primary variable (exercise enjoyment or satisfaction). Statistical heterogeneity was assessed using the chi-square test, and the *I*^2^ value was calculated. Heterogeneity was established as low for *I*^2^=25%, moderate for *I*^2^=50%, and high for *I*^2^=75%. The random effects analysis model was used when the heterogeneity was *I*^2^≥50%, and the fixed effects analysis model was used when the heterogeneity was *I*^2^<50%.

The standardized mean difference (SMD) was used for the overall effect on enjoyment or satisfaction, as different scales were implemented in the included studies. For all enjoyment or satisfaction scales, higher scores implied a better result on this outcome.

For all variables, a statistical significance level of *P*<.05 and 95% CIs were established. The effect size was determined as low when SMD was 0.2, moderate when SMD was 0.5, and high when SMD was 0.8, according to Cohen. Sensitivity analysis was performed individually per study to analyze their influence on the overall results and changes in heterogeneity according to study weight. RevMan software (version 5.4.1; The Cochrane Collaboration) was used for the quantitative analysis.

### Deviations From the Protocol

Some analyses foreseen in the protocol registered in PROSPERO could not be performed in this review. Secondary outcomes were foreseen to meta-analyze the adherence to exercise through video games and the adverse effects that these interventions could have; however, this analysis could not be performed, as adherence and the appearance of adverse effects were measured in only 1 [33] and 2 [33,34] studies, respectively.

## Results

### Study Selection

**Figure 1.**
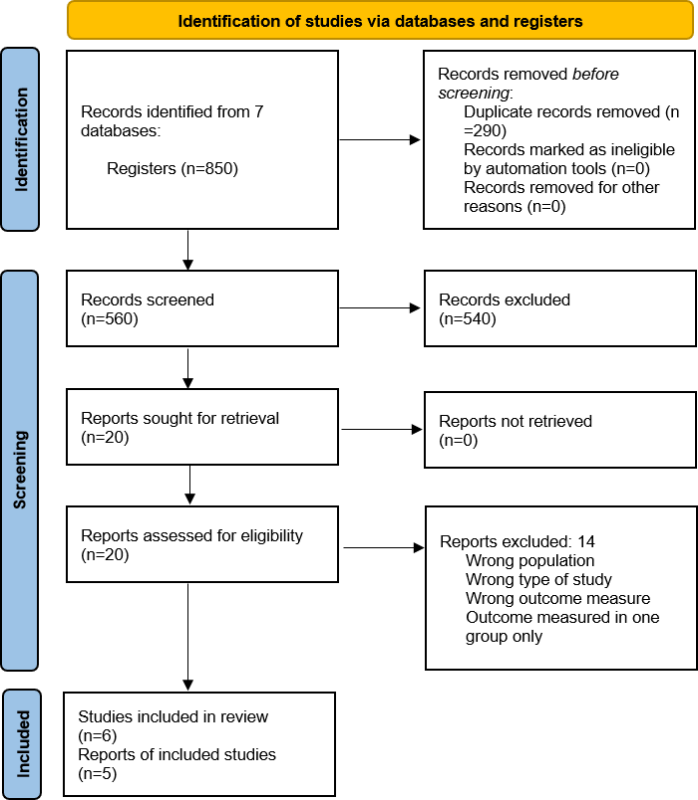
Flow diagram in this review.

A total of 850 studies were retrieved from the search strategy. After the elimination of 290 duplicated papers, 560 papers were screened, of which 540 were excluded after reading the title and abstract. The remaining 20 studies were included for full-text reading, of which only 6 [33-38] were included in this systematic review, as represented in Figure 1. In the quantitative analysis, 5 studies [33,35-38] encompassing a total of 419 participants were included, with a mean age of 74.72 (SD 6.4) years; 215 participants played an active video game, while 204 participants received other interventions. Takei et al [34] were contacted to obtain the unavailable data in their published paper, but no response was received. This selection process underscores the robustness of the study inclusion.

### Characteristics of the Included Studies

This systematic review was performed on 6 studies: 2 randomized controlled trials and 4 crossover studies, involving 419 participants [33-38]. Among these, 3 studies focused on older adults without reported health conditions [35,36,38], 2 on older adults undergoing rehabilitation [33,34], and 1 specifically on older adults with Parkinson disease and mild cognitive impairment [37].

Most studies implemented active video game interventions that incorporated full-body movements, combining various therapeutic approaches such as strength training, balance exercises, flexibility routines, yoga, and jogging [33-36], while 2 studies focused on specific exercises for upper limbs and gait, respectively [37,38].

The comparison groups varied across studies. Dockx et al [37], Sayar et al [36], and Takei et al [34] compared the effects of active video games to those of another active intervention, while Kruse et al [38] and Oesch et al [33] compared the effects of video games to those of videos or exercise leaflets. Ferreira et al’s [35] trial contrasted the effects of active video games with those of watching television. In terms of immersion, 5 studies [33-37] employed augmented reality, while Kruse et al’s [38] study used virtual reality.

Regarding session duration and frequency, training times typically ranged from 30 minutes to 1 hour, except for Kruse et al’s [38] study, which had sessions lasting for 7-10 minutes. Four studies conducted only 1 session of the active video games [34-36,38], while Oesch et al [33] and Dockx et al [37] implemented interventions 2-3 times per week, with overall duration varying between 10 days and 6 weeks.

Two studies [34,36] included exercise intensity parameters measured using the Rate of Perceived Exertion scale, allowing participants to self-regulate the intensity of their interventions. In both cases, participants adjusted their exercise levels based on their own perceptions of effort. Oesch et al [33] mentioned that their exercise intervention was self-regulated, but they did not provide specific data on how this was measured or its effects.

Secondary outcomes such as adherence were investigated only by Oesch et al [33] who reported an overall adherence rate of 85% for both groups. However, the control group showed higher adherence, as the exergame group had more dropouts due to dissatisfaction with the intervention [33]. Regarding adverse effects, Oesch et al [33] and Takei et al [34] reported no adverse effects in their studies. The remaining studies [35-38] did not specify whether any adverse effects occurred. The general characteristics of the included studies and the intervention characteristics in the included studies are shown in Tables1  2, respectively [33-38].

**Table 1.** General study characteristics.

Study ID	Study design	Participants (n)	Age (years), mean (SD)	Pathology	Intervention frequency	Time (min)
Dockx et al [37], 2017	RCT^a^	281 (114M^b^, 167W^c^)	73.75 (6.66)	Older adults without reported health conditions, older adults with mCI^d^, older adults with Parkinson disease	3 days a week for 6 weeks	45
Ferreira et al [35], 2022	Crossover study	32 (15M, 17W)	66.70 (4.98)	Older adults without reported health conditions	1 session	50
Kruse et al [38], 2021	Crossover study	25 (3M, 22W)	81.24 (4.97)	Older adults without reported health conditions	1 session	7‐10
Oesch et al [33], 2017	RCT	54 (29M, 25W)	74.05 (9.25)	Older adults in rehabilitation	Twice a day for 10 days	30
Sayar et al [36], 2023	Crossover study	40 (17M, 23W)	69.60 (4.16)	Older adults without reported health conditions	1 session	30
Takei et al [34], 2023	Crossover study	16 (3M, 13W)	83 (7)	Older adults in rehabilitation	1 session	60

a RCT: randomized controlled trial.

b M: men.

c W: women.

d mCI: mild cognitive impairment.

**Table 2.** Intervention characteristics in the included studies in this review.

Study ID	Experimental group	Control group	Video game type	Hardware	Software	Movement required	Outcomes	Intensity
Dockx et al [37], 2017	Treadmill with augmented reality	Treadmill	AR^a^	Screen for projecting visual content	Screen simulating walking in the street	Gait	USQ^b^	N/A^c^
Ferreira et al [35], 2022	“Your Shape Fitness Evolved” video game	Watch television	AR	Xbox Kinect	“Your Shape Fitness Evolved” (Stack’ em up, zen develop it, pump it, wall breaker, hurricane)	Full-body movement	PACES^d^	N/A
Kruse et al [38], 2021	VR^e^ video game	Exercise video	VR	Valve Index VR headset	Maestro game VR	Upper limbs exercises	IMI^f^	N/A
Oesch et al [33], 2017	Windows Kinect video games from GameUp Project	Exercise leaflet	AR	Windows Kinect	Game up	Full-body movement	Enjoyment (Likert type scale) adherence and adverse effects	Self-regulated
Sayar et al [36], 2023	Xbox Kinect video game	Brisk walking	AR	Xbox Kinect	“Kinect Adventures!” and “Your Shape Fitness Evolved 2012”	Full-body movement	PACES	RPE^g^ (1-10)
Takei et al [34], 2023	Nintendo switch video game	Physical therapy	AR	Nintendo switch, ring fit, and leg sensor	Nintendo switch video games	Full-body movement	Enjoyment (Likert type scale) and adverse effects	RPE (6-20)

a AR: augmented reality.

b USQ: User Satisfaction Questionnaire.

c N/A: not applicable.

d PACES: Physical Activity Enjoyment Scale.

e VR: virtual reality.

f IMI: Intrinsic Motivation Inventory.

g RPE: Rate of Perceived Exertion.

### Risk of Bias in the Included Studies

**Figure 2.**
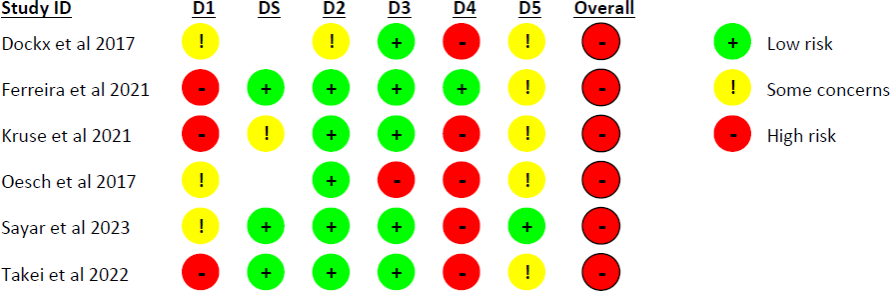
Risk of bias assessment. D1: randomization process. DS: bias arising from period and carryover effects. D2: deviation from intended interventions. D3: missing outcome data. D4: measurement of the outcome. D5: selection of the reported result.

The agreement rate achieved between the two authors who completed the risk of bias assessment was 80%; in case of disagreement (20%), the third reviewer resolved it. The risk of bias in the 6 studies is represented in Figure 2 [33-38].

The Egger regression–based test was conducted to evaluate the presence of publication bias. The results indicated that the intercept was not significantly different from 0 (intercept=2.985; *P*=.83), suggesting no evidence of small-study effects.

**Figure 3.**
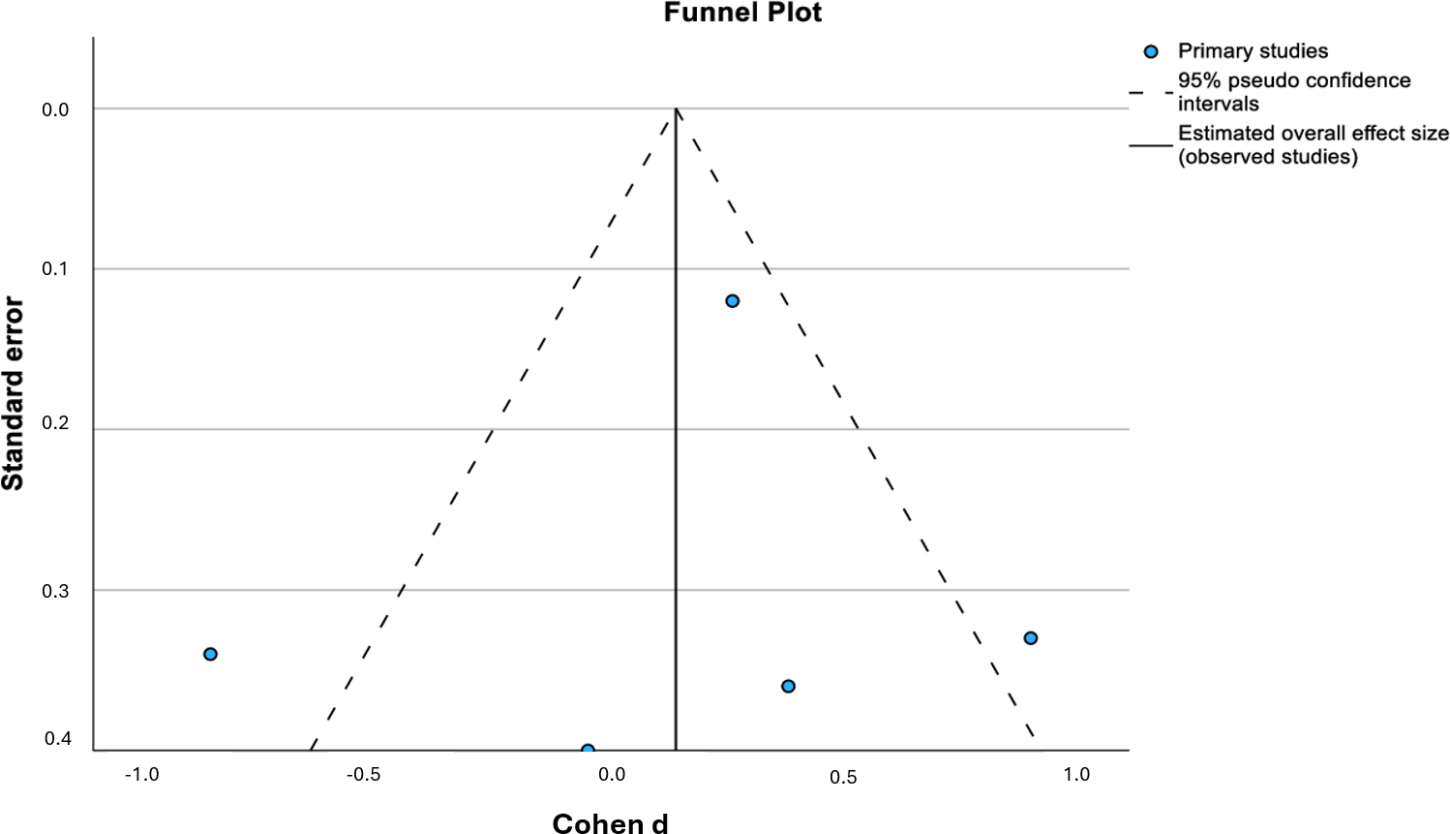
Funnel plot of the included studies.

The funnel plot (Figure 3) visually supports these findings, showing a relatively symmetric distribution of the effect sizes around the estimated overall effect size. The absence of asymmetry further suggests that publication bias is unlikely to have significantly influenced the results of this meta-analysis.

Based on these findings, there is no statistical evidence of publication bias in the included studies.

### Quantitative Analysis

**Figure 4.**
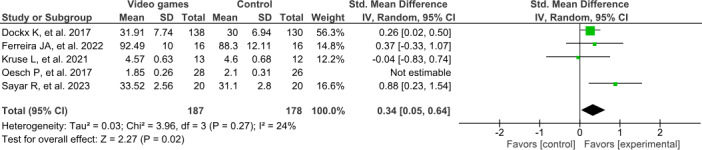
Forest plot of the overall enjoyment.

The active video game group showed an improvement in the overall enjoyment or satisfaction compared to the control group after the intervention period, as shown in Figure 4 [33,35-38], which shows a small effect size and low heterogeneity (SMD 0.34, 95% CI 0.05-0.64; *P*=.02; *I*^2^=24%). The certainty of the evidence for overall enjoyment in active video games versus that in control interventions was rated as low according to the GRADE approach. This was based on data from 4 randomized trials involving 187 participants in the intervention groups and 178 in the control groups. The SMD for enjoyment was 0.34 SD (95% CI 0.05-0.64) higher in the active video game group. The evidence was downgraded due to very serious risk of bias, while inconsistency, indirectness, and imprecision were not considered serious. No other concerns were identified (Multimedia Appendix 2).

For the quantitative analysis, the study by Oesch et al [33] was removed after the sensitivity analysis because it had a small sample size and a large effect size (SMD −0.86, 95% CI −1.42 to −0.3), which increased heterogeneity by 55% for a study weight of 20.6%. Its inclusion significantly affected the overall effect result (SMD 0.12, 95% CI −0.41 to 0.64; *P*=.67; *I*^2^=79%), as shown in Multimedia Appendix 3.

The effect of active video games on exercise enjoyment or satisfaction by subgroups is shown in Table 3. No significant differences were found based on session time, target population, immersion type, number of sessions, or control group type. However, some subgroup comparisons were close to reaching statistical significance. Notably, when the effects of active video games were compared to those of an active intervention, enjoyment was higher with active video games, although this difference was not statistically significant (*P*=.08). Additionally, 2 other subgroup analyses approached significance: older adults without reported health conditions appeared to enjoy active video games more (*P*=.12), and fewer sessions seemed to result in greater enjoyment or satisfaction (*P*=.12).

**Table 3.** Subgroup analysis.

Subgroup	Studies (n)	Participants (n)	Random effect		Heterogeneity (%), *I*^*2*^	Subgroup difference	
			SMD^a^ (95%CI)	*P* value		Chi-square *(df)*	*P* value
Session time (min)						0.1 (1)	0.71
<10	1	25	−0.04 (-−0.83 to 0.74)	0.91	N/A^b^		
>10	4	394	0.15 (−0.48 to 0.78)	0.67	79		
Target population						1.3 (1)	0.25
Older adults without reported health conditionss	3	97	0.44 (−0.09 to 0.96)	0.1	39		
Older adults with reported health conditions	2	322	−0.27 (−1.37 to 0.83)	0.63	92		
Immersion type						0.1 (1)	0.71
Virtual reality	1	25	−0.04 (−0.83 to 0.74)	0.91	N/A		
Augmented reality	4	394	0.15 (−0.48 to 0.78)	0.64	84		
Number of sessions						1.3 (1)	0.25
1	3	97	0.44 (−0.09 to 0.96)	0.1	39		
>10	2	322	−0.27 (−1.37 to 0.83)	0.63	92		
Control group type						1.9 (1)	0.16
Active intervention	2	308	0.49 (−0.1 to 1.09)	0.1	68		
Passive intervention	3	111	−0.21 (−0.98 to 0.57)	0.6	74		

a SMD: standardized mean difference.

b N/A: not applicable.

## Discussion

### Principal Findings

This systematic review with meta-analysis evaluates the specific effectiveness of active video games on enjoyment and satisfaction experienced by older adults—outcomes that are crucial for adherence to physical activity programs. Our findings indicate that exercise delivered through active video games could provide greater enjoyment or satisfaction than control interventions.

Enjoyment is a key determinant in long-term adherence to physical activity, as it enhances engagement and sustainability. Consequently, incorporating enjoyable components such as active video games into exercise regimens aligns with proposals to include fun within the FITT principles for a more holistic exercise prescription [16]. Studies have shown that enjoyment could serve as either a barrier or a facilitator in the adherence to exercise routines [15,26]. Therefore, incorporating enjoyable elements such as active video games into exercise routines or treatments could potentially help individuals stay engaged in a physical activity, as the overall effect on enjoyment and satisfaction in this review suggests that active video games may offer a modest advantage over control interventions, potentially enhancing the appeal of exercise for older participants.

In this systematic review, we found that only Oesch et al [33] examined adherence to active video game interventions, and they reported that the control group showed higher adherence, while the experimental group showed a higher dropout rate due to participants disliking the treatment. These findings contrast with those of Valenzuela et al [24] who demonstrated increased adherence to technology-based interventions. This divergence may stem from differences in the intervention design, participant characteristics, or contextual factors, warranting further exploration. However, the results from Oesch et al’s [33] study may align with general adherence trends for physical activity, where nonadherence rates range from 47% to 96% within the first year in healthy populations and from 50% to 70% in patients undergoing physical therapy, while adherence rates in older adults range from 65% to 86% [26,39-41].

Among the reviewed studies, only Oesch et al [33] and Takei et al [34] reported adverse effects, with neither identifying any incidents during their interventions. Although these findings suggest that active video game interventions are generally safe, the absence of reporting in other studies limits definitive conclusions regarding their safety profiles [33,34]. However, most of the studies [35-38] included did not report the occurrence of adverse effects.

Subgroup analyses revealed no statistically significant differences between active video game interventions and control interventions; however, comparisons between the active video game intervention group and the control group approached significance (*P*=.08). This trend suggests a potential for differential effects that may become apparent with larger sample sizes or more targeted studies. Sayar et al [36] used a crossover design, allowing participants to experience both interventions and compare them directly in terms of enjoyment. This design made it possible to observe which intervention generated a greater sense of enjoyment among participants. In contrast, Dockx et al [37] compared the effects of the usual treadmill walking intervention with those of an intervention that had a screen simulating standard treadmill walking, and they suggested that the added visual and auditory distractions may have contributed to participants’ preference for the screen-enhanced intervention, as participants may have perceived less exertion [42,43].

Other comparisons approaching significance (*P*=.12) were found in older adults without reported health conditions versus older adults with reported health conditions and in 1 session versus >10 sessions, with the same studies [33,35-38] included in each subgroup for the number of sessions. One possible explanation for greater enjoyment in fewer sessions is that repeated exposure to the same intervention might lead to decreased motivation, as older adults could lose interest in the video game or view the technology as more of a barrier than a facilitator [19,24]. Similarly, this may also explain why some older adults with health issues did not favor this type of treatment. A study [44] on older adults experiencing chronic low back pain indicated that continued engagement in physical activity was often due to the enjoyable experience of the exercise itself. In contrast, in Oesch et al’s [33] study, older adults who did not find the activity enjoyable frequently might have perceived it as a barrier, which in some cases contributed to their decision to drop out of the study [33].

### Limitations

This review has several limitations. The generalizability of our findings is limited by the small number of the included studies and their high risk of bias, primarily due to issues in randomization and blinding of participants and assessors. Although the statistical heterogeneity was low after removing one study [33] that significantly increased the variability, differences in the intervention types and outcome measurement methods still contribute to some methodological inconsistencies. Additionally, only 2 studies [34,36] reported on the exercise intensity—a factor known to influence enjoyment and satisfaction through established models [21,45,46]. Future research should explore how exercise enjoyment affects adherence in rehabilitation programs and examine whether perceived exertion influences enjoyment or satisfaction. Addressing these gaps could strengthen the evidence base. Other possible research directions include a systematic review on the role of active video games in adherence or a qualitative study exploring factors that influence older adults’ adherence to exercise programs.

### Clinical Implications

From a clinical perspective, this review does not establish a definitive advantage of active video games over traditional interventions. However, tailoring exercise programs to individual preferences and integrating enjoyable elements may optimize patient adherence and satisfaction, aligning with patient-centered care principles.

### Conclusion

Active video games could help improve enjoyment or satisfaction in older adults, with a low certainty of evidence. In this systematic review, active video games did not show a superior effect to conventional treatment on adherence. Future research should explore optimizing gamification techniques to maximize adherence and satisfaction.

## Supplementary material

10.2196/72559 Multimedia Appendix 1 Search strategy.

10.2196/72559 Multimedia Appendix 2 Assessment of evidence according to GRADE (Grades of Recommendation Assessment Development and Evaluation).

10.2196/72559 Multimedia Appendix 3 Forest plot of the overall enjoyment, including Oesch et al’s [33] study.

10.2196/72559 Checklist 1 PRISMA (Preferred Reporting Items for Systematic Reviews and Meta-Analyses) checklist.

## Abbreviations

FITT: Frequency, Intensity, Time, and Type
GRADE: Grades of Recommendation Assessment Development and Evaluation
PICOS: Population, Intervention, Comparison, Outcome
PRISMA: Preferred Reporting Items for Systematic Reviews and Meta-Analyses
SMD: standardized mean difference
WHO: World Health Organization
