# Supplementary material for: Effectiveness of Gamification on Enjoyment and Satisfaction in Older Adults: Systematic Review and Meta-Analysis
Source: JMIR Aging. 2025 Jun 12;8:e72559. doi: 10.2196/72559 (PMC12178586; doi:10.2196/72559)
Supplement: Multimedia Appendix 1 [file aging-v8-e72559-s001.docx]

PUBMED

("virtual reality"[MeSH] OR "exergaming"[MeSH] OR "data display"[MeSH] OR "video games"[MeSH] OR "augmented reality"[MeSH] OR "extended reality" OR "serious games" OR "digital game" OR "active video game" OR "motion captured" OR "motion based" OR "gamified exercise") AND ("Patient Satisfaction"[Mesh] OR "satisfaction" OR "enjoyability" OR "enjoyment") AND ("aged"[MeSH] OR "nursing homes"[MeSH] OR "older adults" OR "senior" OR "elderly" OR "institutionalized" OR "care home" OR "long-term care" OR "assisted living" OR "resident*" OR "residential facilities") Filter: Clinical trials

COCHRANE

("virtual reality"[MeSH] OR "exergaming"[MeSH] OR "data display"[MeSH] OR "video games"[MeSH] OR "augmented reality"[MeSH] OR "extended reality" OR "serious games" OR "digital game" OR "active video game" OR "motion captured" OR "motion based" OR "gamified exercise") AND ("Patient Satisfaction"[Mesh] OR "satisfaction" OR "enjoyability" OR "enjoyment") AND ("aged"[MeSH] OR "nursing homes"[MeSH] OR "older adults" OR "senior" OR "elderly" OR "institutionalized" OR "care home" OR "long-term care" OR "assisted living" OR (NEXT resident*) OR "residential facilities") 🡪Filter: Trials

CINHAL

AB ("aged" OR "nursing homes" OR "older adults" OR "senior" OR "elderly" OR "institutionalized" OR "care home" OR "long-term care" OR "assisted living" OR "resident*" OR "residential facilities") AND AB ("Patient Satisfaction" OR "satisfaction" OR "enjoyability" OR "enjoyment") AND AB ("virtual reality" OR "exergaming" OR "data display" OR "video games" OR "augmented reality" OR "extended reality" OR "serious games" OR "digital game" OR "active video game" OR "motion captured" OR "motion based" OR "gamified exercise") Filer: Academic research

SPORT DISCUS

AB ("aged" OR "nursing homes" OR "older adults" OR "senior" OR "elderly" OR "institutionalized" OR "care home" OR "long-term care" OR "assisted living" OR "resident*" OR "residential facilities") AND AB ("Patient Satisfaction" OR "satisfaction" OR "enjoyability" OR "enjoyment") AND AB ("virtual reality"[MeSH] OR "exergaming" OR "data display" OR "video games" OR "augmented reality" OR "extended reality" OR "serious games" OR "digital game" OR "active video game" OR "motion captured" OR "motion based" OR "gamified exercise") Filer: Academic research

WOS

("virtual reality" OR "exergaming" OR "data display" OR "video games" OR "augmented reality" OR "extended reality" OR "serious games" OR "digital game" OR "active video game" OR "motion captured" OR "motion based" OR "gamified exercise") AND ("Patient Satisfaction" OR "satisfaction" OR "enjoyability" OR "enjoyment") AND ("aged" OR "nursing homes" OR "older adults" OR "senior" OR "elderly" OR "institutionalized" OR "care home" OR "long-term care" OR "assisted living" OR "resident*" OR "residential facilities") Filter: Clinical trial

SCOPUS

TITLE-ABS ( ( "virtual reality" OR "exergaming" OR "data display" OR "video games" OR "augmented reality" OR "extended reality" OR "serious games" OR "digital game" OR "active video game" OR "motion captured" OR "motion based" OR "gamified exercise" ) AND ( "Patient Satisfaction" OR "satisfaction" OR "enjoyability" OR "enjoyment" ) AND ( "aged" OR "nursing homes" OR "older adults" OR "senior" OR "elderly" OR "institutionalized" OR "care home" OR "long-term care" OR "assisted living" OR "resident*" OR "residential facilities" ) ) Filter: Article

PEDRO

"older adults" AND exergaming AND enjoyment 🡪1

elderly AND exergaming AND enjoyment 🡪0

"older adults" AND video games AND enjoyment 🡪 0

"older adults" AND video games AND patient satisfaction🡪 0
