# Supplementary material for: Effectiveness of Gamification on Enjoyment and Satisfaction in Older Adults: Systematic Review and Meta-Analysis
Source: JMIR Aging. 2025 Jun 12;8:e72559. doi: 10.2196/72559 (PMC12178586; doi:10.2196/72559)
Supplement: Multimedia Appendix 2 [file aging-v8-e72559-s002.docx]

| **Certainty assessment** | | | | | | | **№ of patients** | | **Effect** | | **Certainty** | **Importance** |
| --- | --- | --- | --- | --- | --- | --- | --- | --- | --- | --- | --- | --- |
| **№ of studies** | **Study design** | **Risk of bias** | **Inconsistency** | **Indirectness** | **Imprecision** | **Other considerations** | **Active videogames** | **control interventions** | **Relative (95% CI)** | **Absolute (95% CI)** |  |  |
| **Overall enjoyment videogames vs control interventions** | | | | | | | | | | | | |
| 4 | randomised trials | very serious^a^ | not serious^b^ | not serious^c^ | not serious^d^ | none | 187 | 178 | - | SMD **0.34 SD higher** (0.05 higher to 0.64 higher) | ⨁⨁◯◯ Low^a,b,c,d^ | IMPORTANT |

***Table 2:*** Assessment of evidence according to GRADE. CI: Confidence interval; SMD: Standardized mean difference. (a) The studies present a high risk of bias. (b) Heterogeneity <25%. (c) No publication bias detected (Egger test p>0.05). (d) Heterogeneity <75%.
