# Supplementary figures and images for: Effectiveness of Gamification on Enjoyment and Satisfaction in Older Adults: Systematic Review and Meta-Analysis
Source: JMIR Aging. 2025 Jun 12;8:e72559. doi: 10.2196/72559 (PMC12178586; doi:10.2196/72559)

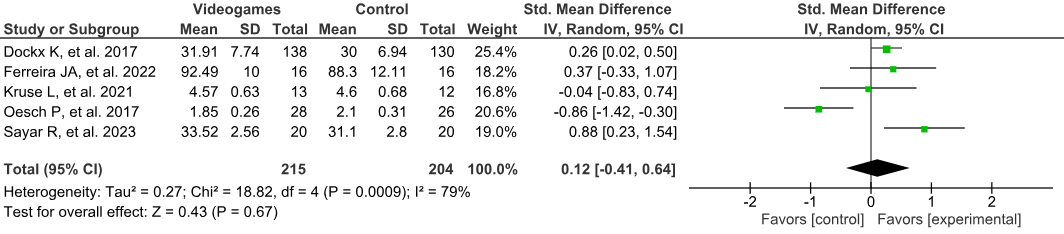

Supplement: Multimedia Appendix 3 [file aging-v8-e72559-s003.png]
